# Linking multiple biodiversity informatics platforms with Darwin Core Archives

**DOI:** 10.3897/BDJ.2.e1039

**Published:** 2014-01-08

**Authors:** Ed Baker, Simon Rycroft, Vincent S Smith

**Affiliations:** † Natural History Museum, London, United Kingdom

**Keywords:** Darwin Core, Darwin Core Archive, Scratchpads, biodiversity informatics, virtual research environment, taxonomy, biodiversity

## Abstract

We describe an implementation of the Darwin Core Archive (DwC-A) standard that allows for the exchange of biodiversity information contained within the Scratchpads virtual research environment with external collaborators. Using this single archive file Scratchpad users can expose taxonomies, specimen records, species descriptions and a range of other data to a variety of third-party aggregators and tools (currently Encyclopedia of Life, eMonocot Portal, CartoDB, and the Common Data Model) for secondary use. This paper describes our technical approach to dynamically building and validating Darwin Core Archives for the 600+ Scratchpad user communities, which can be used to serve the diverse data needs of all of our content partners.

## Introduction

Biodiversity data are frequently published in esoteric formats or described using non-standard terminology, lacking sufficient contextual information to facilitate reuse, or a technical mechanism to facilitate data exchange. The Darwin Core Archive (DwC-A) format which is based on the Darwin Core standard (Wieczorek et al. 2012), helps to address this problem. DwC-A's have become the *lingua franca* of the biodiversity science community, acting as a bridging language to facilitate the exchange of biodiversity data between systems storing and managing biodiversity data. This standardisation, which supports methods of data citation, not only encourages data sharing through appropriate reward mechanisms, but also supports the integration of data from multiple sources. This enables more complex scientific questions to be addressed from multiple data sources, without manual processing of the data structure, format or contextual metadata. This community-level harmonization and interoperability has the potential to prevent community projects from becoming data silos. Shared data-exchange standards like DwC-A make it possible to aggregate data in community ‘staging posts’, merging them in various combinations, perform meta-analyses and submitting them to public repositories.

Scratchpads (Smith et al. 2011) are an example of an online tool for communities to collect, curate and publish biodiversity data. With over 7,000 active users in 600 different research communities, generating data on more than 180,000 taxa, Scratchpad data are potentially of great use to many biodiversity data aggregators and services such as the Encyclopedia of Life (EoL) and the Global Biodiversity Informatics Facility (GBIF). These staging posts allow communities to build and curate biodiversity data sets of any size for their own purpose, while contributing to larger efforts or making use of tools that support the DwC-A format. As part of the EU funded ViBRANT project we have used the DwC-A format to facilitate data exchange across a variety of projects and tools constructed by the 17 partners within the ViBRANT consortium. Similarly the NERC funded eMonocot project has a requirement to aggregate data from communities of monocot specialists working within their Scratchpads. These data need to be aggregated within a central eMonocot portal that integrates the data with other sources and provide cross-cutting views of the data.

This paper describes our technical solution to implement a single DwC-A format to exchange data with a variety of external systems. This contrasts with previous methods of data reuse using DwC-A that have typically been developed for a single common dataset and intended for reuse by a single partner (e.g. Remsen et al. 2012). Our approach in the Scratchpads reduces the code base and processing power required to ensure that all of our external partners can be served from a single DwC-A file. However, our implementation has been complicated by the varied requirements of our collaborators and the multiple ways in which some Scratchpad communities have recorded data within their Scratchpads. In particular, it has exposed variation in the way DwC-A files can be written and interpreted, and in the testing procedures of some third party projects that only validate portions of DwC-A files relevant to their specific project activities or constraints. In an effort to overcome these problems, we describe our attempts to accommodate the variation in the interpretation of DwC-A files and a new DwC-A validator, which works across the diversity of data compiled by many Scratchpad communities.

## Project description

### Funding

This project was funded, and uses infrastructure developed by the European Union funded ViBRANT project (Contract no. RI-261532) and the Natural Environment Research Council funded eMonocot project (Grant numbers 279981, 279984 & 27997).

## Web location (URIs)

Download page: https://git.scratchpads.eu/v/scratchpads-2.0.git/tree/HEAD:/sites/all/modules/custom/dwca_export

Bug database: http://support.scratchpads.eu/projects/eol/issues

Vendor: http://scratchpads.eu

## Technical specification

Platform: Scratchpads/Drupal

Programming language: PHP

Language: English

Service endpoint: [scratchpads.url]/dwca.zip

## Repository

Type (Git, SVN, CVS, Arch, BK): Git

Browse uri (CVS, SVN, BK): https://git.scratchpads.eu/v/scratchpads-2.0.git/tree/HEAD:/sites/all/modules/custom/dwca_export

## Usage rights

### Use license

Other

### IP rights notes

The code for this project is licensed under the GNU General Public License, version 2.

## Implementation

### Implements specification

**Figure 1.**
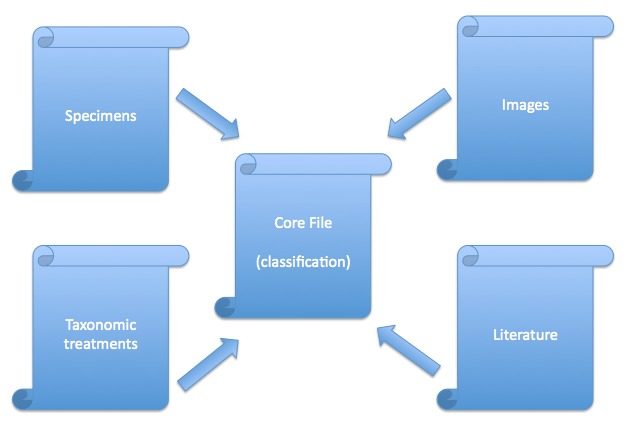
The star schema showing the relation of Darwin Core Archive extension files to the core file.

The Darwin Core Archive standard is a human and machine readable system for exchanging information on biological diversity. It consists of a central data file (e.g. a taxonomy) and any number of extensions (e.g. specimens, images) that point to the central file (a star schema, see Baker, 2013 & Fig. 1). As the data in Scratchpads are predominately about taxa (images of taxa, descriptions of taxa, specimens of taxa,...) we have chosen to use the Scratchpad's taxonomic classification as our central file. The extensions that we use cover descriptions of taxa using the Species Profile Model, the distribution of taxa, images and other media, associated literature references, specimens, vernacular names, the users of the site and comments made on site content. The content of the files may be described using Darwin Core terms, or terms from other schemas. These data files are all plain text (UTF-8) files.

In addition to the data files each archive has a meta.xml file that defines what the fields are in each data file, how rows and fields are terminated in each file, and what type of data are contained in each file (rowType). Of particular interest to our implementation is the fact that for a particular file in the archive the meta.xml can define multiple meanings by using different rowTypes. For example this allows us to treat descriptions.txt as both TDWG Descriptions (as defined at http://rs.gbif.org/terms/1.0/Description) and EoL Media items (as defined at http://eol.org/schema/media/Document). More information on this implementation is in the description.txt section below.

**Figure 2.**
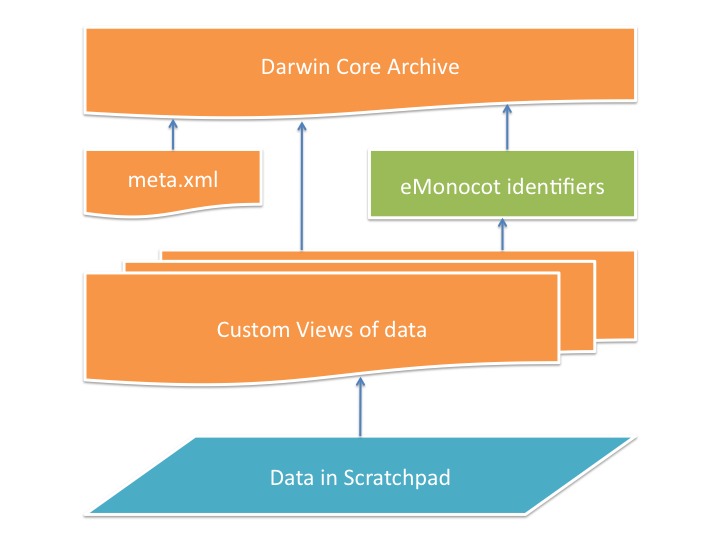
Data flow from Scratchpad to Darwin Core Archive. The dwca_export module (orange) defines a number of custom Drupal Views (queries) that collect the data required for archive generation from the Scratchpad (blue) and combines them with the meta.xml which describes the information in the archive. For eMonocot Scratchpads the emonocot_dwca module (green) provides an intermediary function replacing the Scratchpads internal unique identifiers with those used throughout the eMonocot project (see eMonocot modifications section).

The Drupal module we have written, dwca_export (Fig. 2), provides new default Drupal views (see Views) that match the structures we have specified in our meta.xml file. The Views data export module provides the functionality to export these views as comma-separated values text files. This module also provides the functionality to create these text files in a batch operation (appending several rows at a time to the output file to reduce memory usage) from the command line. This batch operation reduces the memory required to produce the archive, as early implementations of this module for large exports required 1GB of memory, which is a large amount of resource in a shared hosting environment. In most cases one view is used to produce one text file in the DwC-A. The exception is the description.txt which uses one view for each Species Profile Model (SPM) field. A list of the SPM fields we use is at https://git.scratchpads.eu/v/scratchpads.git/blob/695831817b318a4eef42a31de1e61bd6f67145a7:/sites/all/modules/spm/EOL_SPM.xml. The output of these views is concatenated in a later stage of the archive creation process.

To conserve server resources the DwC-As are generated as a background process, triggered by a change being made to the content of the site. It is also possible for the Scratchpad administration team to rebuild the archives on demand using Drush via the command line (see Drush section below).

### Audience

The functionality provided by this module can be enabled by the maintainer on any Scratchpad site through the Tools page found at Admin > Structure. Once enabled, the archive file will become available at [scratchpads.url]/dwca.zip after it has been created by the system.

This module is for Scratchpad maintainers who wish to share the content of their site with third party aggregators.

## Additional information

### Biological Classifications (classification.txt)

The classification.txt file forms the core of our star schema and is consistent between all third party users.

Scratchpads as a system are designed to facilitate classifications conforming to any (or no) code of nomenclature. While we change the Scratchpad interface to match the terminology of a particular code on a per-classification basis, Darwin Core standardises the terminology used for the taxonomic and nomenclatural status of a taxon (http://rs.gbif.org/vocabulary/gbif/taxonomic_status.xml, http://rs.gbif.org/vocabulary/gbif/nomenclatural_status.xml). It is therefore necessary for us to map from the ontologies we use (derived from ITIS, the Integrated Taxonomic Information System) to the DwC standards. This mapping is shown in Table 2.

The field used are shown in Table 1.

### Taxon Descriptions (description.txt)

Descriptions used for describing the identification and biology of taxa in Scratchpads are defined using the TDWG Species Profile Model. A precise definition of the fields that we support is available from the relevant part of the Scratchpads Git repository and match the fields used by EoL in the Lifedesks project.

**Figure 3.**
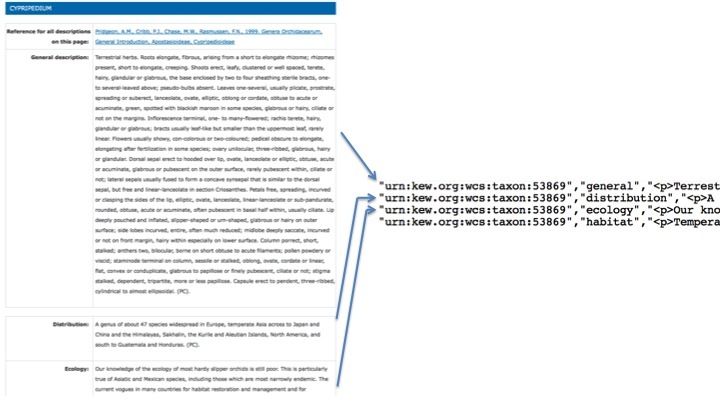
A single Taxon Description node on a Scratchpad corresponds to one or more rows in the description.txt file.

In Scratchpads it is possible to create one or more taxon description nodes for every taxon. These nodes allow entry of text into any of the fields described above. In a DwC-A each of these fields is considered a record in itself (Fig. 3). For this reason the universally unique identifier of a Taxon Description (the Scratchpad node identifier) is not enough to uniquely identify each row in the descriptions.txt file. To address this we append the hash (#) character and the field name to the end of the node UUID to create an additional identifier column for projects such as eMonocot which need it.

The way that the eMonocot Portal and EoL handle textual descriptions of taxa and narratives on taxon biology is very different and our implementation needs to accommodate both. The eMonocot portal ingests these data using the GBIF Taxon Description extension to DwC-A whereas EoL expects them to be treated as media items associated with a taxon using their own media extension (http://eol.org/schema/media/Document). In order to fulfil our aim of creating a single archive for all consumers we need to provide different definitions of the descriptions file in the meta.xml. For this reason in the meta.xml we define two different rowTypes for the same file (Table 3) – this allows the consumer to choose the definition they prefer without the Scratchpads having to create separate Darwin Core Archives.

### Content Authors (eol_agents.txt)

The EoL agents file is a non-standard (not conforming to the star schema) extension to DwC-A that records information about people who have contributed to the other content in the archive (e.g. authors of textual content, the photographer of images). As this rowType is only understood by EoL the presence of this file in the archive does not cause problems for other consumers of the DwC-A. The structure of this file is described in Table 4.

### Comments on content (comment.txt – for eMonocot use)

For the eMonocot project there is a need for comments on data in the portal and comment replies in the Scratchpad to be synchronised across both platforms. This synchronisation was implemented using the DwC-A, ensuring that this one export mechanism could serve all of the project's needs. This extension to the archive does not conform to the star schema as it does not link directly to the central classification.txt. Despite this the rowType is, at present, only understood by eMonocot, and is ignored by other projects reading the archive. The data model used is a subset of the Open Annotation Data Model (http://www.w3.org/ns/oa#Annotationt). The structure of this file is described in Table 5.

### references.txt

This file contains information from the Scratchpad site's bibliography. The structure of the file is described in Table 6.

### specimen.txt

This table contains the museum/herbarium specimens and observation records present within a Scratchpad. At present Scratchpads use DwC 1.2.1 as the internal specimen/observation standard. This is currently being upgraded to DwC 1.4. Table 7.

### vernacular_names.txt

The vernacular names extension records common names, their language, where in the world they are used and any remarks (Table 8).

### eMonocot modifications to the DwC-A

The eMonocot project makes extensive use of its own unique identifiers derived from the World Checklist of Monocots (WCM). A separate Scratchpads/Drupal module, emonocot_dwca, handles the replacement of the Scratchpads UUID with the eMonocot/WCM identifier. In addition, the licences for taxon descriptive content from eMonocot sites are derived through a link to a publication in the Scratchpad bibliography. A licence field on that publication is used to assign a licence to all the descriptive content that has been copied from that resource. This function is not present in standard Scratchpads, and the emonocot_dwca module ensures that the correct licence is applied to content when it is exported in the DwC-A.

### Usage of Drush (for use by Scratchpad Team or server administrator)

One additional Drush command is provided by the module dwca_rebuild. This function needs to be called twice to build an archive. The first time it is called it creates a number of processes run via the POSIX nohup command (POSIX, the Portable Operating System Interface, is a set of standards for Unix-like operatinng systems) to prevent problems with timeouts when connected to a server via SSH. These processes run through the individual views used to create the DwC-A files and output their contents to text files. The second time it is run the various description views are aggregated into a single file, and this, along with the other exported text files are then zipped to create the archive.

This feature is used by the Scratchpads Team (or the server administrator where the site is not hosted by the Scratchpad project) and is not available to Scratchpads users.

#### Example usage

From within a site directory: drush dwca_rebuild

Using an alias: drush @cypriioideae.e-monocot.org dwca_rebuild

### Module requirements

This module has dependencies on a number of standard Drupal contributed modules (Views, Views Data Export) and also the Darwin Core module developed for the Scratchpads project. While the module may run correctly on a non-Scratchpad Drupal installation this is not a supported use. The module can be enabled on a Scratchpad site through the Structure > Tools interface, which will automatically enable all dependencies.

### DwC-A Validator

A number of tools exist that can perform validation on a DwC-A file, for example the tools developed by EoL and GBIF. While these tools are useful they are insufficient for complete verification of an archive. The EoL validator is specifically designed to interpret content destined for the Encyclopedia of Life project, and as such, only reports errors when, for example, licences incompatible with EoL's terms and conditions are used, although in this case there is no requirement for using an EoL licence in the definition of a DwC-A. The EoL validator also reports errors when an image is listed multiple times in the media extension, a situation that may arise when a photograph on a Scratchpad site contains two identifiable species. In contrast the GBIF validator checks for basic compliance with the schema (testing structure rather than content), and no validator is available for the eMonocot project.

In order to provide a consistent and correct DwC-A from a Scratchpad to any of our content partners the decision was made to create our own validator. This validator checks the structure of the archive, ensuring that all files and columns specified in the meta.xml are present in the zip file. Where columns should contain structured content (e.g. a URL, ISSN, ISBN) the content in each row is checked for conformity using regular expression matching. A complete list of checks currently performed is available on the validator wiki.

Checking the structure of the file allows us to check for errors such as column transposition during any future development to the dwca_export module. The precise checking of data in each row also allows us to check the data in the Scratchpad. This gives us the potential to add a feature that warns users of potential errors in the content present within their Scratchpad. For example, running the validator against a number of sites identified several occasions where ISSNs had incorrectly been entered into the ISBN field. At present the validator is run as a stand-alone tool – future work will provide Scratchpad maintainers with a list of identified errors with links to edit content flagged by the validator.

### Usage of archives

Once the module is enabled the Darwin Core Archive is available for use by our content partners in ViBRANT and eMonocot as well as by anybody else who has an interest in the data. Users of the data must abide by the licence (generally Creative Commons) that the site maintainer has specified. It should be noted that individual rows of data are likely to have different licences.

### Content of archives

By default all of the content in the site that is available to the public via the standard web interface is available in the archive. Content creators can hide content from both the Scratchpad website and the Darwin Core Archive by marking that content as 'Unpublished'. It should be noted that due to the caching of web pages and the archives being created by a cron task that content may take a few hours to disappear from both the website and the archive.

At present only content types specified by the Scratchpad team (Specimens, Taxonomy, References, Images, etc.) can be mapped into the Darwin Core Archive. In the future we plan to provide an interface to allow custom content types created by site maintainers to be mapped into the archive.

## Figures and Tables

**Table 1.** The fields used in our classification.txt – the core of our DwC-A star schema.

Field	Description	Term
Taxon ID	A universally unique identifier (UUID) of this name (World Checklist of Monocots [WCM] identifier for eMonocot Scratchpads)	
Taxon Name	The taxon name – made by concatenating the unit names and unit indicators (the Scratchpads stores all parts of the scientific name, and indicators such as sp. & spp. in separate fields)	http://rs.tdwg.org/dwc/terms/scientificName
Taxonomic Status	See Table 2	http://rs.tdwg.org/dwc/terms/taxonomicStatus
Taxonomic Rank	e.g. species, genus, family	http://rs.tdwg.org/dwc/terms/taxonRank
Taxon Author(s)	Plain text names of the author(s) of this taxon	http://rs.tdwg.org/dwc/terms/scientificNameAuthorship
Reference	Citation of the reference containing the description of this taxon	http://rs.tdwg.org/dwc/terms/namePublishedIn
Reference ID	URL to the reference containing the description of this taxon within the Scratchpad	http://rs.tdwg.org/dwc/terms/namePublishedInID
Taxonomic Parent	The parent of this name in the classification, if this name is accepted.	http://rs.tdwg.org/dwc/terms/parentNameUsageID
Nomenclatural Status	See Table 2	http://rs.tdwg.org/dwc/terms/nomenclaturalStatus
Accepted Name	The UUID of the associated accepted name, if this name is not accepted	http://rs.tdwg.org/dwc/terms/acceptedNameUsageID

**Table 2.** Mapping from the Scratchpads taxonomy model to the GBIF Darwin Core taxonomy model

Scratchpads: Usage	Scratchpads: Unacceptability Reason	DwC: taxonomicStatus	DwC: nomenclaturalStatus
accepted/valid		accepted	
valid		valid	
not accepted / invalid	-None-		
not accepted / invalid	synonym	synonym	
not accepted / invalid	homotypic (nomenclatural) synonym	homotypicSynonym	
not accepted / invalid	heterotypic (taxonomic) synonym	heterotypicSynonym	
not accepted / invalid	homonym (illegitimate)	heterotypicSynonym	illegitimum
not accepted / invalid	superfluous renaming (illegitimate)	homotypicSyonym	superfluum
not accepted / invalid	rejected name	synonym	rejiciendum
not accepted / invalid	invalidly published, nomen nudum	synonym	nudum
not accepted / invalid	invalidly published, other	synonym	invalidum
not accepted / invalid	misapplied	misapplied	
not accepted / invalid	pro parte	proParteSynonym	
not accepted / invalid	horticultural		
not accepted / invalid	database artifact		
not accepted / invalid	orthographic Variant (misspelling)	synonym	orthographia
not accepted / invalid	other		
not accepted / invalid	junior synonym		
not accepted / invalid	objective synonym		
not accepted / invalid	subjective synonym		
not accepted / invalid	original name/combination		
not accepted / invalid	subsequent name/combination		combinatio
not accepted / invalid	junior homonym	synonym	illegitimum
not accepted / invalid	homonym & junior synonym	synonym	
not accepted / invalid	unavailable, database artifact		
not accepted / invalid	unavailable, literature misspelling	synonym	orthographia
not accepted / invalid	unavailable, incorrect original spelling		negatum
not accepted / invalid	unavailable, suppressed by ruling		oppressa
not accepted / invalid	unavailable, nomen nudum	synonym	nudum
not accepted / invalid	unavailable, other		
not accepted / invalid	unjustified emendation		
not accepted / invalid	unnecessary replacement	synonym	superfluum
not accepted / invalid	nomen oblitum		
not accepted / invalid	misapplied		
not accepted / invalid	pro parte	proParteSynonym	
not accepted / invalid	nomen dubium	synonym	dubium

**Table 3.** Mapping schema of Scratchpads textual descriptions of taxa to the GBIF (as used by eMonocot) and EoL extensions in the Darwin Core Archive.

Field	Description	GBIF term	EoL term
Taxon ID	The Scratchpads universally unique identifier for the taxonomic name (WCM identifier for eMonocot Scratchpads)	http://rs.tdwg.org/dwc/terms/taxonID	http://rs.tdwg.org/dwc/terms/taxonID
Type	The type of the textual description: "general", "ecology", "behaviour", etc.	http://purl.org/dc/terms/type	
Text	The textual description. E.g. if Type is "general" this field will contain a general description of the taxon	http://purl.org/dc/terms/description	http://purl.org/dc/terms/description
Rights	Textual description of the rights associated with this content, e.g. "All Rights Reserved"	http://purl.org/dc/terms/rights	
AccessURI	The URL of the Scratchpad node containing this description	http://purl.org/dc/terms/references	http://rs.tdwg.org/ac/terms/furtherInformationUR
Source	Scratchpad URL of the bibliographic reference this description is from (only applies to eMonocot sites)	http://purl.org/dc/terms/source	
Copyright Owner	The copyright owner of the description		http://ns.adobe.com/xap/1.0/rights/Owner
Language	The language of the description	http://purl.org/dc/terms/language	http://purl.org/dc/terms/language
CV Term	Image keywords		http://iptc.org/std/Iptc4xmpExt/1.0/xmlns/CVterm
Format	URL of the SPM type of the description, e.g. http://rs.tdwg.org/ontology/voc/SPMInfoItems#GeneralDescription		http://purl.org/dc/terms/format
Type	MIME type of the textual content, generally: text/html		http://purl.org/dc/terms/type
agentID	UUID of the author who contributed the content to the site		http://eol.org/schema/agent/agentID
License	URL of the license used to release the content, if any	http://purl.org/dc/terms/license	http://ns.adobe.com/xap/1.0/rights/UsageTerms
Identifier	A unique identifier for this particular textual description. Formed by concatenating the universally unique identifier of the description node, the # character, and the field name.	http://purl.org/dc/terms/identifier	http://purl.org/dc/terms/identifier

**Table 4.** eol_agents

Field	Description	Term
User ID	The universally unique identifier of the user	http://purl.org/dc/terms/identifier
Family Name	The user's last/family name	http://xmlns.com/foaf/spec/#term_familyName
First Names	The user's given/first names	http://xmlns.com/foaf/spec/#term_firstName
Full Name	Full name of the user – concatentaion with space of above two fields	http://xmlns.com/foaf/spec/#term_name
Organisation	The organisation the user works for, if any	http://eol.org/schema/agent/organization
Username	The user's username on this Scratchpad	http://xmlns.com/foaf/spec/#term_accountName

**Table 5.** Structure of comments.txt – the non-standard extension for synchronising comments between a Scratchpad and the eMonocot portal

Field	Description	Term
CommentID	URL of the comment	http://purl.org/dc/terms/identifier
Target	URL of the node the comment was made on	http://www.w3.org/ns/oa#hasTarget
Title	Title of the comment	http://purl.org/dc/terms/title
Body	The comment itself	http://www.w3.org/ns/oa#hasBody
Created	Date and time the comment was created	http://purl.org/dc/terms/created
Modified	Date and time the comment was last edited	http://purl.org/dc/terms/modified

**Table 6.** references

Field	Description	GBIF / PURL Term	EoL Term
Taxon ID	UUID of taxa in the publication		
Identifier	UUID of the reference in the Scratchpad site		http://purl.org/dc/terms/identifier
DOI	Digital Object Identifier	http://purl.org/ontology/bibo/doi	http://purl.org/ontology/bibo/doi
ISBN	International Standard Book Number	http://purl.org/ontology/bibo/isbn	
ISSN	International Standard Serial Number	http://purl.org/ontology/bibo/issn	
Citation	Plain text citation of the work	http://purl.org/dc/terms/bibliographicCitation	http://eol.org/schema/reference/full_reference
Title		http://purl.org/dc/terms/title	http://eol.org/schema/reference/primaryTitle
		http://purl.org/dc/terms/creator	
Node URL		http://purl.org/dc/terms/identifier	
		http://purl.org/dc/terms/subject	
Language		http://purl.org/dc/terms/language	http://purl.org/dc/terms/language
	Indicates if if publication is original description of a taxon	http://purl.org/dc/terms/type	
Publication Date		http://purl.org/dc/terms/date	
Created Date	Date the reference was added to the Scratchpad	http://purl.org/dc/terms/created	
Modified Date	Date the reference was last modified in the Scratchpad	http://purl.org/dc/terms/modified	
		http://purl.org/dc/terms/relation	
		http://purl.org/dc/terms/source	

**Table 7.** specimens.txt

Field	Description	Term
Taxon ID		
Type Status	e.g. Holotype	http://rs.tdwg.org/dwc/terms/typeStatus
Institution Code	e.g. BMNH for Natural History Museum, London	http://rs.tdwg.org/dwc/terms/institutionCode
Collection Code	e.g. E for Entomology	http://rs.tdwg.org/dwc/terms/collectionCode
Catalogue Number	Unique specimen identifier	http://rs.tdwg.org/dwc/terms/catalogNumber
Latitude	Decimal latitude	http://rs.tdwg.org/dwc/terms/decimalLatitude
Longitude	Decimal longitude	http://rs.tdwg.org/dwc/terms/decimalLongitude

**Table 8.** vernacular_names.txt

Field	Description	Term
Taxon ID		
Vernacular Name	The vernacular (common) name	http://rs.tdwg.org/dwc/terms/vernacularName
Language	The language of the vernacular name	http://purl.org/dc/terms/language
Locality	Where is the vernacular name used	http://rs.tdwg.org/dwc/terms/locality
Remarks	Other information	http://rs.tdwg.org/dwc/terms/taxonRemarks

## Supplementary files

### Sample Darwin Core Archive from eMonocot Scratchpad

**Authors:** eMonocot Cypripedioideae **Data type:** darwin core archive Darwin Core Archive created from the eMonocot Cypripedioidea Scratchpad on 06/01/2014. **File:** oo_5522.zip
